# The Late Dr. Marshall H. Webb

**Published:** 1883-06

**Authors:** E. Osmond


### The Late Dr. Marshall H. Webb.

REPLY TO DR. JAS. W. WHITE.

The review by Dr. Jas. W. White, editor of the *Dental Cosmos*, May number, of my article on the late Dr. Marshall H. Webb, in the April number of this journal (which he reprints) demands a reply. He calls my article "an impertinence" If criticising a firm, which has for years sought to lord it over our profession, is an impertinence, this impertinent individual will ask another question. "Upon what meat doth this our Ceasar feed, that he has grown so great."

It is hard to say which should be most admired, their *modesty*

in withholding their arrangement with the late Dr. Webb from the public, or the *very loud advertisement* they have given it *since* the publication of my article would, of course, never have been written had the facts (?) in the case been before made public by them.

The readers of the *Cosmos* are invited to compare the paragraph begining, "The intimation that Dr. Webb neglected," &c., with that commencing, "He was constantly in receipt of," &c., and they will be able to form their own conclusions. Like the lawyer who pleaded his own case, editor White has told too much.

It is not necessary to reply to the expressions, "stirring up the mud in certain dirty pools," questioning the respectability of this journal, "personal animus," "spleen, &c.," a malicious fling at a business-house over a dead man's coffin, as they are merely the coarse utterances of an angry man. But, as regards the last expression, I will suggest to him, that if he will provide coffins for all the different patents his firm has bought up and *buried*, simply because they conflicted with their *business interests*, and might have been developed to the *good of our profession* by other firms, it will keep him busy for some time. That this policy was the settled one of the late Founder of the house, is well known, a man, whose many virtues and services to our profession, entitle his memory to the highest respect, but who, like the rest of us, had his human failings. In this connection we may quote the words of Shakespeare.

"The evil that men do, lives after them ;
The good is oft interred with their bones."

E. Osmond, M.D., D.D.S.

